# Adverse Effects of Classical Swine Fever Virus LOM Vaccine and Jeju LOM Strains in Pregnant Sows and Specific Pathogen-Free Pigs

**DOI:** 10.3390/pathogens9010018

**Published:** 2019-12-23

**Authors:** SeEun Choe, Jae-Hoon Kim, Ki-Sun Kim, Sok Song, Ra Mi Cha, Wan-Choul Kang, Hyeun-Ju Kim, Gyu-Nam Park, Jihye Shin, Hyoung-Nam Jo, In-Soo Cho, Bang-Hun Hyun, Bong-Kyun Park, Dong-Jun An

**Affiliations:** 1 Viral Disease Division, Animal and Plant Quarantine Agency (APQA), Gimcheon, Gyeongbuk 39660, Korea; ivvi59@korea.kr (S.C.); kisunkim@korea.kr (K.-S.K.); ssoboro@naver.com (S.S.); rami.cha01@korea.kr (R.M.C.); changep0418@gmail.com (G.-N.P.); shinjibong227@gmail.com (J.S.); chois38@korea.kr (I.-S.C.); hyunbh@korea.kr (B.-H.H.); park026@korea.kr (B.-K.P.); 2 College of Veterinary Medicine and Veterinary Medical Research Institute, Jeju National University, Jeju 63243, Koreaengle59@naver.com (H.-N.J.); 3 Jeju Special Self-Governing Provincial Veterinary Research Institute, Jeju 63344, Korea; kwc1041@korea.kr (W.-C.K.); bluemouse@korea.kr (H.-J.K.); 4 College of Veterinary Medicine, Seoul University, Gwanak-ro, Gwanak-gu, Seoul 08826, Korea

**Keywords:** CSFV, pathogenicity, MLV-LOM, SPF pig, Jeju LOM strain

## Abstract

In Jeju island of South Korea, a classical swine fever (CSF) non-vaccinated region, many pig farmers insisted on abortion and stillbirth in pregnant sows and high mortality of suckling/weaning piglets by circulating CSF virus from 2014 to 2018. We investigated whether CSF viruses isolated from pigs in Jeju Island (Jeju LOM) have recovered their pathogenicity by conducting experiments using pregnant sows and specific pathogen-free (SPF) pigs. The CSF modified live LOM vaccine (MLV-LOM) and Jeju LOM strains induced abortion and stillbirth in pregnant sows. Viral antigens were detected in the organs of fetuses and stillborn piglets in the absence of specific pathological lesions associated with the virulent CSF virus in both groups (MLV-LOM and Jeju LOM strain). However, antigen was detected in one newborn piglet from a sow inoculated with a Jeju LOM strain, suggesting that it may cause persistent infections in pigs. SPF pigs inoculated with the MLV-LOM or Jeju LOM strains were asymptomatic, but virus antigen was detected in several organ and blood samples. Virus shedding in both groups of animals was not detected in the feces or saliva until 21 days post inoculation. The serum concentration of the three major cytokines, IFN-α, TNF-α, and IL-10, known to be related to lymphocytopenia, were similar in both groups when the MLV-LOM or Jeju LOM strains were inoculated into SPF pigs. In conclusion, Jeju LOM strains exhibited most of the characteristics of the MLV-LOM in pigs and resulted in the same adverse effects as the MLV-LOM strain.

## 1. Introduction

Classical swine fever virus (CSFV) is a small, enveloped virus with a positive-stranded RNA genome of approximately 12.5 kb in size and contains a single, large, open reading frame that encodes a 3898 amino acid (aa) polyprotein [1]. Classical swine fever (CSF) is a devastating disease, causing substantial economic losses through the death of valuable livestock. Major outbreak of CSF is now rare, but sporadic epizootic incidents still occur frequently, causing chronic, atypical forms of the disease. Virulent CSFV enters the host through the mucous membranes of the oral and nasal cavities, known to infect tonsil cells and then spread into whole body using the circulation (blood and lymph) systems [2]. When CSFV infects pregnant sows, vertical transmission to the fetus was reported, which means CSFV may infect the fetus via passing placental barrier and the affected fetus may carry persistent infection (PI) [2]. CSFV-infected sows, depending on the gestation stage, show mild clinical signs whereas infection may result in mummification or absorption of fetuses and ends pregnancy with abortions or stillbirth [3,4,5,6,7].

Acute CSFV infection may result in high fever, leukopenia, thrombocytopenia, and hemorrhages in various organs [8]. CSFV also caused multiple pathological lesions such as enlarged lymph nodes, hemorrhages, and petechiae on the serosal and mucosal surfaces of many organs including the lungs, kidneys, intestines, and urinary bladder [8]. Previous reports showed that acute CSF infection induces a so-called cytokine storm by aberrant levels of type I interferon (IFN) and proinflammatory mediators [8,9]. It has been shown that lymphocyte depletion is related to a strong IFN-α response [9] and lymphocytopenia is closely associated with interleukin IL-1α, IL-6, and tumor necrosis factor TNF-α responses [10]. Chronic CSFV infection known to cause pathological changes including atrophy of the thymus, depletion of the lymphoid organs, necrosis, and ulceration of the small intestine, colon, and ileocecal valve [8]. These clinical signs and lesions were also caused by many other swine pathogens [8]. Various non-specific clinical signs and lesions among animals may be due to the host factor and the virulence of the CSFV strain. In addition, age, breed and immune status of each animal often play a role in the outcome of disease [11,12,13].

Use of a CSF-modified live LOM (Low virulence strain of Miyagi) vaccine (MLV-LOM) in pigs in Korea since 1974 was shown to result in adverse effects, such as abortion and stillbirth, in naïve pregnant sows that had not produced CSF antibody [14]. When the MLV-LOM was inoculated into piglets already infected with immunosuppressive pathogens, the MLV-LOM induced vaccine-specific antibodies without any adverse effects. [15]. However, the MLV-LOM remained in the pigs’ bodies for a longer time, reflecting the possibility that virus shedding and transmission might occur [15]. A previous report showed that oral administration of the MLV-LOM can induce immunity in pigs. [16]. 

The Korean government maintains a MLV-LOM policy for CSF control on the mainland but not on Jeju Island, where the MLV-LOM vaccine has not been used since 2000 [17]. In 2014, pregnant sows on 20 Jeju Island pig farms were inoculated to commercial swine erysipelas vaccine mixed with the MLV-LOM [17]. Administration of the vaccine was stopped immediately, but between 2015 and 2018, pigs on an additional 91 farms were found to have been transmitted to the virus [17]. Jeju farmers and some pig disease experts insisted on abortion and stillbirth of pregnant sows and high mortality of suckling/weaning piglets by Jeju LOM strain [17]. Therefore, the safety of the MLV-LOM was questioned and its reversion to pathogenicity suspected.

The main purpose of this study was to determine whether the Jeju LOM strains on Jeju Island exhibited pathogenic properties when inoculated into pregnant sows and specific pathogen-free (SPF) pigs. We also analyze the causes of MLV-LOM strain influx and spread in CSF non-vaccinated area.

## 2. Results 

### 2.1. Viral Antigen Detection in Pregnant Pigs 3 Weeks Post Inoculation

One (no. 37-5952) of three pregnant sows in group 1 was autopsied three weeks post inoculation (wpi) with strain JJ16LOM-YJK08 (Jeju LOM), and CSFV RNA was detected in the tonsils by qRT-PCR. In addition, 17/20 (85%) fetuses of the same pregnant sow were positive for CSFV RNA by qRT-PCR (Table 1). CSFV RNA was detected in the heart of a pregnant sow (no. 51-2104) in group 2, which was inoculated with strain JJ16LOM-YYM02 (Jeju LOM), and 9/14 (64%) fetuses were CSFV RNA positive (Table 1). In a pregnant sow (no. LI-5534) in group 3, inoculated with strain 16LOM-KM00 (MLV-LOM), a tonsil sample was CSFV RNA positive, and 7/9 (78%) fetuses were positive for the CSFV RNA (Table 1). No histologic preparations of the three pregnant sows revealed lesions associated with CSF. Pathologic examination of the internal organs of 43 fetuses from the three sows showed no specific lesions, and no viral antigen was detected by the immunohistochemical (IHC) assay. 

### 2.2. Pathogenicity in Pregnant Pigs and Their Piglets

In group 1, inoculated with strain JJ16LOM-YJK08 (Jeju LOM), and group 2, inoculated with strain JJ16LOM-YYM02 (Jeju LOM), the total number of fetuses produced was 23 and 32, respectively. In group 1, nine fetuses were mummified, 13 were stillborn, and one was a live birth; in group 2, 11 were mummified, 15 were stillborn, and six were live births. The crown–rump (C-R) length range of fetuses was 5.0–28.5 cm for group 1 and 6.5–30.0 cm for group 2 (Table 2). The total number of fetuses in group 3 inoculated with strain 16LOM-KM00 (MLV-LOM) were 13, of which 12 were mummified and one was a live birth. The C-R length of fetuses ranged from 11.5 to 28 cm (Table 2). CSFV RNA detection in fetuses was 20/23 (86.9%) for group 1, 23/32 (71.8%) for group 2, and 12/13 (92.3%) for group 3 samples by qRT-PCR (Table 2). When strains JJ16LOM-YJK08 and JJ16LOM-YYM02 were used as inocula, CSFV RNA was detected in all samples (13/13 and 15/15, respectively) from stillborn fetuses, and all 12 mummified fetuses in group 3 contained CSFV RNA (Table 2). In addition, one out of eight live piglets tested from all groups (1–3) was CSFV RNA positive in organ samples (Table 2). 

**Figure 1:**
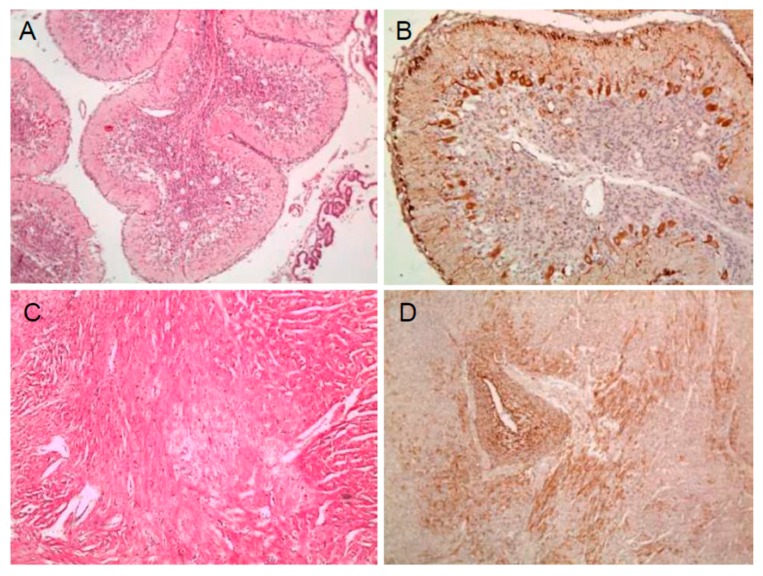
Severe vacuolation in the white matter of cerebellum of a weak born piglet (**A**); severe myocardial necrosis in the heart of a weak born piglet (**C**); and expression of LOM antigens in cerebellum (**B**) and heart (**D**) (A and C: H & E, × 200) and (B and D: IHC, × 200).

One weak-born piglet and one still-born fetus delivered from sow no. 34-5053 in group 1 exhibited necrosis, hemorrhage, severe vacuolation, and perivascular cuffing in the white matter of cerebellum, and one fetus also exhibited severe myocardial necrosis in the heart (Figure 1A,C). Three piglets from sow no. 62-0093 in group 2 were identified as non-suppurative encephalitis with hemorrhage and mineral deposition in the white matter of in the cerebellum. However, no specific lesions were observed in fetuses in the other groups (3 and 4). By IHC staining, viral antigens were detected in the internal organs group 1 weak born piglet and stillborn fetus (Figure 1B,D) and in the heart of one stillborn group 2 fetus. Seroconversions for CSF neutralizing antibodies in pregnant pigs were on average 7 log _2_ for group 1, 6.5 log _2_ for group 2, and 7 log _2_ for group 3, when measured 3 wpi, and 7 log _2_, 9.5 log _2_, and 10 log _2_ at delivery, respectively.

### 2.3. Pathogenicity in SPF Pigs 

**Figure 2:**
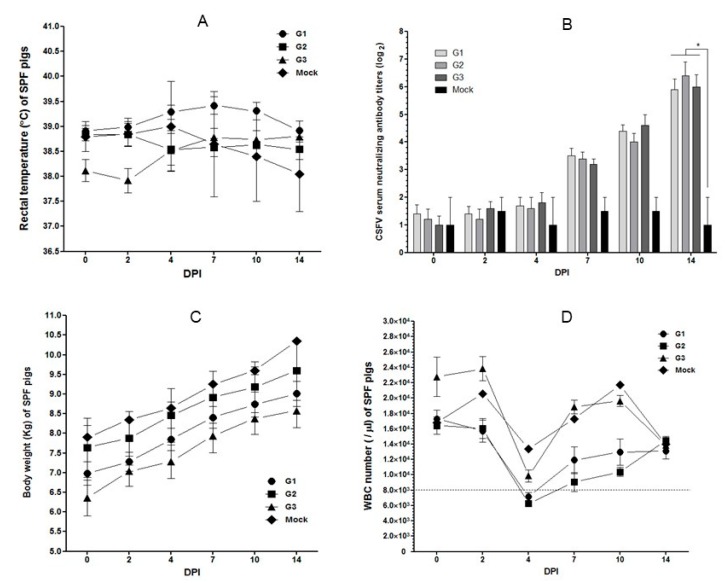
Changes in rectal temperature, neutralization antibodies, bodyweights, and white blood cell counts of specific pathogen-free pigs inoculated with the JJ16LOM-YJK08 in G1, JJ16LOM-YJK-F in G2, and 16LOM-KO00 in G3. Rectal temperature (**A**), neutralization antibodies (**B**), bodyweight (**C**), and white blood cells (**D**). Bars represent the mean ± standard error (SE) during animal experiment period. * *p* < 0.05 compared with neutralization antibody values among groups at 14 dpi (**B**). Below dotted line indicate leukopenia (**D**).

The rectal temperatures of 22 SPF pigs did not exceed 40 °C until 14 days post inoculation (Figure 2A). CSF neutralizing antibodies for SPF pigs at 14 days post inoculation (dpi) were on average 5.9 log _2_ for group 1 inoculated with strain JJ16LOM-YJK08 (Jeju LOM), 6.4 log _2_ for group 2 inoculated with strain JJ16LOM-YJK08-F (Jeju LOM), and 6.0 log _2_ for group 3 inoculated with strain 16LOM-KO00 (MLV-LOM) (Figure 2B). In all groups, the neutralizing antibody titers showed a significant seroconversion (*p* < 0.05) when compared with uninoculated control group 4 at 14 dpi (Figure 2B). Virus-inoculated and uninoculated SPF pigs gained average weights of 2.02, 1.96, 2.22, and 2.45 kg in groups 1, 2, 3, and 4, respectively, during the observation period (14 days; Figure 2C). White blood cell (WBC) counts in the SPF pigs in groups 1, 2, 3, and 4 decreased to averages of 7142, 6300, 9868, and 13,400/µl at 4 dpi, respectively (Figure 2D). At 4 dpi, 6/10 SPF pigs (60%) in group 1 and 5/5 pigs (100%) in group 2 showed symptoms of temporary leukopenia (Figure 2D). 

### 2.4. Histopathogenic Lesions and Virus Shedding in SPF Pigs 

For group 1 SPF pigs, CSFV RNA was detected in 6/10 (60%) blood samples at 7 dpi, 8/10 (80%) at 10 dpi, and 3/10 (30%) at 14 dpi by qRT-PCR (Table 3). For group 2 pigs, CSFV RNA was detected in 4/5 (80%) blood samples at 7 dpi, 5/5 (100%) at 10 dpi, and 2/5 (40%) at 14 dpi. For group 3 SPF pigs, CSFV RNA was detected in only 2/5 (40%) blood samples tested at 10 dpi (Table 3). However, CSFV RNA was not detected in the saliva or feces of any of the SPF pigs tested (Table 3). 

**Figure 3:**
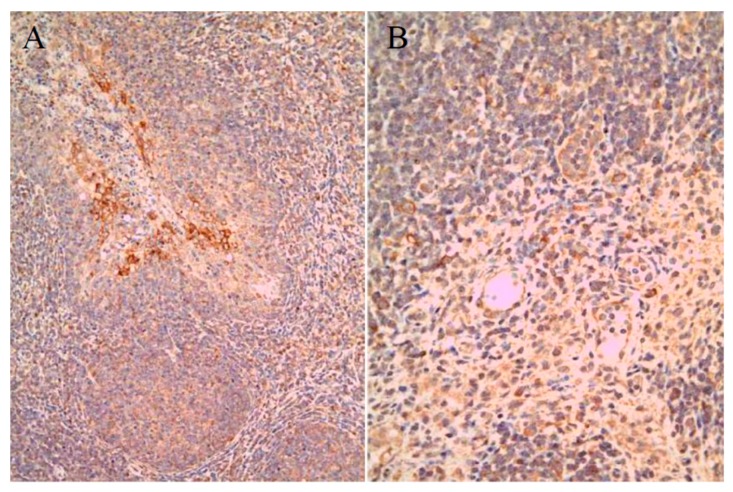
Immunohistochemical (IHC) results in SPF pigs. LOM antigens in the cryptal epithelium of tonsil (**A**) and solitary mono-nuclear cells in lymph node (**B**). Magnification × 200.

By IHC staining, viral antigens were detected within the organs of 10 group 1 SPF pigs, specifically in 7/10 (70%) tonsil, 1/10 (10%) spleen, 1/10 (10%) ileum, and 2/10 (20%) submandibular lymph node samples (Figure 3, Table 4). In addition, SPF pigs in group 2 were positive in 3/5 (60%) tonsil samples only, while samples from group 3 SPF pigs contained antigens in all samples, i.e., 5/5 (100%) tonsil samples, 2/5 (40%) spleen samples, and 1/5 (20%) ileum sample (Table 4).

### 2.5. Cytokine Concentrations in SPF Pigs Inoculated with the MLV-LOM and Jeju LOM Strains 

**Figure 4:**
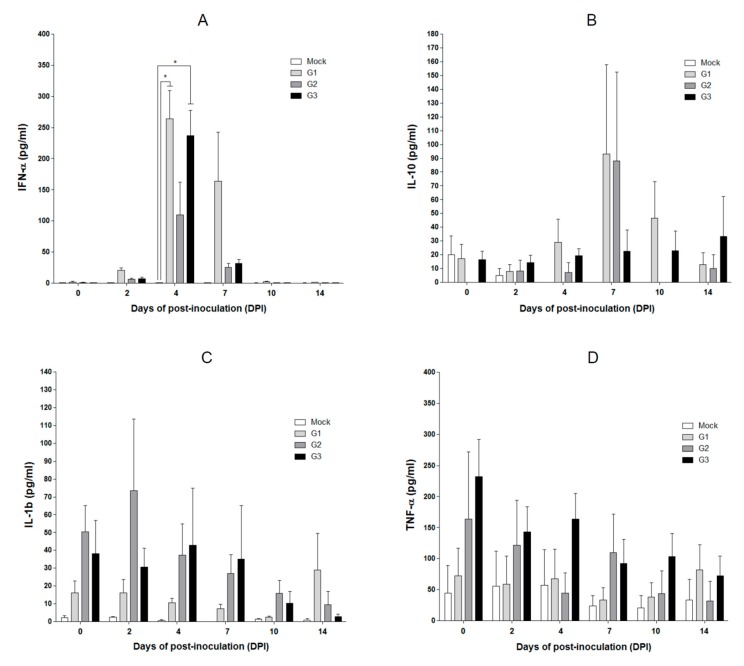
Cytokine concentrations in serum of SPF pigs over time post-infection. IFN-α (**A**), IL-10 (**B**), IL-1β (**C**), and TNF-α (**D**). Results are presented as mean ± standard deviation (SD) between 0 dpi and 14 dpi. Mock group is marked as the white column. G1 (JJ16LOM-YJK08), G2 (JJ16LOM-YJK08-F), and G3 (16LOM-KO11) are marked light grey, dark grey, and black, respectively. * *p* < 0.05 (A).

**Figure 5:**
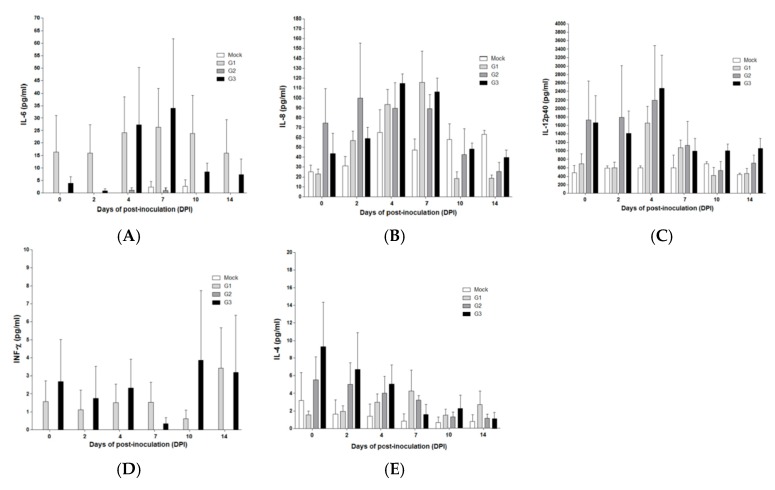
Cytokine levels in post-infection sera in SPF pigs. IL-6 (**A**), IL-8 (**B**), IL-12p40 (**C**), IFN-γ (**D**), and IL-4 (**E**). Results are presented as mean ± standard deviation (SD) between 0 dpi and 14 dpi. Mock group is marked as a white column. G1 (JJ16LOM-YJK08), G2 (JJ16LOM-YJK08-F), and G3 (16LOM-KO11) are marked light grey, dark grey, and black, respectively.

The concentrations of IFN-α in sera were similar in SPF pigs in groups 1 (JJ16LOM-YJK08) and 3 (16LOM-KO11) at 4 dpi, but the concentration was higher in group 1 pigs at 7 dpi (Figure 4A). IL-10 was higher in group 1(JJ16LOM-YJK08) and 2 (JJ16LOM-YJK08-F) SPF pigs (Figure 4B) at 7 dpi. In addition, IL-1β concentrations were higher at 2 dpi in group 2 SPF pigs than in other groups (Figure 4C). The remaining cytokines (TNF-α, IL-6, IL-8, IL-12p40, IFNγ, and IL-4) showed no significant differences during the observation period between SPF pigs inoculated with the three strains (JJ16LOM-YJK08, JJ16LOM-YJK08-F, and 16LOM-KO11) (Figure 4D and Figure 5A–E).

## 3. Discussion

The immunogenicity of the CSF MLV-LOM in pregnant sows was found to be excellent, but it was detected in some organs of their fetuses and sows, thus confirming that the MLV-LOM can pass through the placenta and infect fetuses [14]. In addition, the occurrence of stillborn or mummified fetuses suggests that the MLV-LOM may retain some pathogenicity in gestating pigs [14]. Interestingly, our results showed that the overall prevalence of infected fetuses was higher (92.3%) with the MLV-LOM than with the Jeju LOM strains (71.8–86.9%) isolated from Jeju Island. Histopathological examination revealed degeneration and necrosis of myocytes in the heart. Non-suppurative encephalitis and multifocal mineralizations in the brain only occurred in 9.1% (5/55) of fetuses delivered from sows inoculated with Jeju Island isolates. In addition, viral antigens were detected by the IHC test in 5.5% (3/55) of fetuses. One live piglet delivered from a sow inoculated with strain JJ16LOM-YJK08 exhibited severe vacuolar change with perivascular cuffing in cerebellum and myocardial degeneration, and a large amount of antigen was expressed in internal parenchymal organs. If the piglet had not been autopsied, it would have remained persistently infected and act as a carrier, steadily releasing the antigen. It has been shown that CSF infection occurs between 50 and 70 days of pregnancy, an immunotolerance phenomenon can be induced, and persistently infected offspring are born [3,4,5,6,7]. These piglets were healthy and survive for several months but die due to late onset form of CSF and they also shed high viral loads, which are enough for transmission to other pigs [3,4,5,6,7]. Recent studies have suggested that persistent infection can occur when newborn piglets are infected within the first 8 h of life, or even up to 48 h after birth [18,19].

In a previous report from 1990, safety tests with SPF pigs were conducted for the LOM-850 strain (the original MLV-LOM) and the LOM-suri strain cloned from LOM-850 [20]. Gross findings in SPF pigs inoculated with both strains were not observed, but all the groups showed mild lesions in the lymph nodes and bladder [20]. At necropsy, 10 days after vaccination, the redness of the lymph nodes was decreased, and bladder bleeding ceased [20]. In addition, IHC assay on SPF pigs at 21 days after vaccination were normal. Overall, gross and pathological findings decreased with time after vaccination and returned to normal at 21 days after vaccination [20]. In this study, we observed no specific clinical symptoms related to CSF in the pathogenicity tests on SPF pigs during the experimental period. However, CSFV RNA was detected in lymphoid organs such as tonsils, lymph nodes, spleen, and ileum, and showed a tendency to decrease in tissues with time post inoculation when assessed by qRT-PCR. When samples of SPF pig lymphoid organs were tested using the IHC stain, no lesions of lymphoid depletion, which are known to be characteristic of virulent CSFV infections, were observed. There was no specific high-temperature reaction in SPF pigs observed in this study, but transient leucopenia was observed in SPF pigs inoculated with strain JJ16LOM-JYK08 or JJ16LOM-JYK08-F at 4 dpi. However, the average WBC levels for these groups were 7142 and 6300/µ1, which were not very low, and later, the WBC levels recovered. 

Lymphocytopenia was well known as one of the typical characteristics of CSFV infections [8,21,22,23]. In general, lymphocytopenia, as well as blood coagulation disorder, are considered to be related to the upregulation of inflammatory cytokines, chemokines, and adhesion factors [24,25]. IFN-α is known to be involved in the induction of lymphocytopenia [9,10], and IL-10 is also known to related to general immunosuppression [26,27]. The concentration of IFN-α was highest at 4 dpi after strain LOM16-KO00 and JJ16LOM-YJK08 inoculation, but the difference between the two was not significant. The concentration of IL-10 at 7 dpi was higher in SPF pigs inoculated with strain JJ16LOM-YJK08 or JJ16LOM-YJK08-F than in those inoculated with LOM16-KO00. The recent study showed that TNF-α and IFN-α expressions in the sera of pigs infected with a moderately virulent CSFV increased, whereas concentrations in pigs following inoculation of the vaccine C-strain remained normal [28]. Generally, TNF-α is believed to be involved in the inflammatory response [28]. It has been also shown that an increase in TNF-α or IFN-α may induce the inflammatory response or apoptosis of lymphocytes after virulent CSFV infection [28]. In addition, TNF-α has been considered, alongside IL-1, IL-6, and IL-4, to be responsible for, among others, apoptosis in immune system cells, such as macrophages [10,29,30] and dendritic cells in lymphoid organs [27]. Several previous reports revealed that the increased expression of IL-8 after infection, without elucidation of its direct role in CSFV pathogenesis [24,25,31]. Our results suggest that TNF-α, IL-4, IL-6, and IL-8 showed no difference during the observation period between the three strains (JJ16LOM-YJK08, JJ16LOM-YJK08-F, and 16LOM-KO11). 

Cytokine concentrations were at their maximum 2–3 days earlier in pigs infected with CSFV strains of moderate and high virulence when compared with those infected with a strain of low virulence [28]. These findings suggest that the C-strain vaccine virus replicates very slowly in some tissues, including tonsils, and therefore escape recognition by host antiviral responses. This slow virus (CSF vaccine strain) replication may have resulted in avoiding the untimely over-expression of IFNs and proinflammatory cytokines, and so indirectly protect immune cells and organs [28]. Based on previous suggestions, we assume that the MLV-LOM strain may share similarities with the C-strain vaccine virus because they are both live attenuated vaccine strains. In conclusion, the CSFV Jeju LOM strains showed no pathogenicity in SPF pigs but exhibited similarly to the MLV-LOM. The Jeju LOM strains produced similar adverse effects in the fetuses of pregnant sows to those of the MLV-LOM. Therefore, we suggest that the Jeju LOM strains are not pathogenic revertants of the MLV-LOM. Our epidemiologic results for CSFV (Jeju LOM) outbreak in Jeju from 2014 to 2018 indicated five major factors that may cause severe damage by Jeju LOM strain in Jeju pig farms of CSF non-vaccinated area. First, inoculation of MLV-LOM strain in pregnant sow (contraindication to vaccination policy); second, no stamping out for first Jeju LOM inoculated pregnant sow and their offspring suckling piglets; third, failure to clean and disinfect feces containing Jeju LOM strain in the pig farms; fourth, failure of disease prevention between slaughterhouse and pig farm; fifth, quarantine failure between mainland (South Korea) and Jeju island. Therefore, the MLV-LOM strain including Jeju LOM strain should never be inoculated in pregnant pigs without anti-CSF antibody. We suggest that MLV-LOM should be replaced quickly with CSF live marker vaccine with excellent safety and efficacy as a government CSF preventive policy. 

## 4. Materials and Methods

### 4.1. Strains, Neutralization Antibody, IHC, and qRT-PCR

The MLV-LOM used was strain 16LOM-KM00. Three Jeju LOM isolates from Jeju Island were designated as strains JJ16LOM-YJK08, JJ16LOM-YJK08-F, and JJ16LOM-YYM02 [17]. A serum neutralization peroxidase-linked antibody (NPLA) assay to detect anti-CSFV antibody and a CSFV qRT-PCR and IHC to detect CSF antigen were analyzed using pig samples (serum and tissue) according to the previous paper [17]. 

### 4.2. Pregnant Pigs Inoculated with MLV-LOM and Jeju LOM Strains

Ten pregnant sows (64–67 days of gestation) were divided into three groups, and were inoculated with strain JJ16LOM-YJK08 (Jeju LOM) in group 1 (n = 3), strain JJ16LOM-YYM02 (Jeju LOM) in group 2 (n = 3), and strain 16LOM-KO00 (MLV-LOM) in group 3 (n = 3) with 10^3.5^ TCID_50_/dose. Group 4 (n = 1) served as an uninoculated negative control. For groups 1–3, one pregnant sow from each group was autopsied at 3 wpi, and the remaining sows and their offspring piglets were autopsied after delivery. To investigate the presence of neutralizing antibodies, a NPLA was performed on the serum of sows collected before inoculation, 3 wpi, and at delivery (7 wpi). At 3 wpi, autopsies of pregnant sows and fetuses were tested for signs of CSF. At delivery, all piglets were examined for farrowing and the presence of antigen in their fetuses. The organs of their piglets were subjected to pathologic analysis. 

### 4.3. SPF Pigs Inoculated with the MLV-LOM and Jeju LOM Strains

Twenty-two SPF pigs (on average 45–50 days old) were purchased from Optifarm company (Osong, Korea) and divided into four groups to allow comparison of the relative pathogenicity of the Jeju LOM and MLV-LOM. Group 1 animals (n = 10) were inoculated with strain JJ16LOM-YJK08 (Jeju LOM). Those in group 2 (n = 5) were inoculated with strain JJ16LOM-YJK-F (Jeju LOM). Those in group 3 (n = 5) were inoculated with a commercial vaccine strain (16LOM-KO00) (MLV-LOM), and group 4 pigs (n = 2) were mock as a negative uninoculated control. Clinical symptoms in SPF pigs were observed daily. Blood, saliva, and fecal swab samples were taken, and weight and temperature checks were performed before inoculation and at 2, 4, 7, 10, and 14 dpi for virus detection, leukocyte counts, weight and temperature changes, and seroconversion. At 14 dpi, five SPF pigs in group 1, five in group 3, and one in group 4 were autopsied to test for the presence of viruses within organs by qRT-PCR and IHC assays, and to note any lesions in tissues. The remaining SPF pigs were autopsied and collected samples (blood, saliva, and fecal) at 21 dpi.

### 4.4. Multiplex Immunoassay for SPF Pigs 

The porcine cytokine and chemokine 9-plex Porcine ProcartaPlexTM Panel 1 (ThermoFisher Scientific, Cat no. EPX090-60829-901) was used to detect nine cytokines and chemokines (IFN-α, IFN-γ, TNF-α, IL-1β, IL-4, IL-6, IL-8, IL-10, and IL-12p40) in the sera from SPF pigs inoculated with MLV-LOM (16LOM-KO11) and Jeju LOM strains (JJ16LOM-YJK08 and JJ16LOM-YJK08-F). ProcartaPlex immunoassays are based on the principles of a sandwich ELISA, using two highly specific antibodies binding to different epitopes of one protein to quantitate all protein targets simultaneously using Luminex^®^ 200^TM^ (Luminex Co., TX, USA). In brief, serum fractions from blood collected in EDTA-containing tubes were obtained after centrifugation at 1000× *g* for 10 min at 20–25 °C. Magnetic beads were vortex-mixed for 30 s, 50 µl of the beads was added to each well, and then pig-specific universal assay buffer and sample were each added in 25 µl volumes to the wells. The plates were shaken at room temperature (RT) for 30 min, overnight at 4 °C in the dark, and then at RT for a further 30 min. Beads were then washed twice. Detection Antibody mix (25 µl) was added to the beads, and the beads were incubated with shaking at RT for 30 min and then washed twice. Streptavidin-PE (50 µl) was then added, and the beads were incubated with shaking at RT for 30 min and washed twice. Reading buffer (120 µl) was added, and incubation continued with shaking at RT for 5 min. The samples were read on Luminex^®^ 200^TM^ (Luminex Co., TX, USA).

### 4.5. Statistical Analysis

The data was analyzed by one-way ANOVA, which was followed by Tukey’s multiple-comparison test using GraphPad Prism software (version 6.0). Results in groups are expressed as mean ± standard error (SE) and significant difference (*p* < 0.05) are indicated by an asterisk.

## Figures and Tables

**Table 1:** Autopsy results for pregnant sows three weeks after inoculation with the modified live (MLV)-LOM or Jeju LOM strains.

Group	Inoculum Strain	No. of Pregnant Sow	Day of Inoculation Post-Pregnancy	Day of Autopsy Post-Pregnancy	Antigen Detection in Organs from Pregnant Sows				Antigen Positive Fetuses/Total Fetuses (%)
					Tonsil	Heart	Lymph Node	Other Organs *	
G1	JJ16LOM-YJK08	37-5952	66	87	+	-	-	-	17/20 (85)
G2	JJ16LOM-YYM02	51-2104	64	85	-	+	-	-	9/14 (64.2)
G3	16LOM-KM00	LI-5534	67	88	+	-	-	-	7/9 (77.7)

* Lung, spleen, liver, kidney, ileum, brain, bladder.

**Table 2:** Neonatal piglets from sows inoculated with MLV-LOM or Jeju LOM strains.

Group	Inoculum Strain	No. of Sow	* Day of Inoculation Post-Pregnancy (Days)	Total Period of Pregnancy (Days)	No. Viral Antigen Positive/No. of Offspring				** Positive Antigen/Test No. (%)	Crown-Rump (cm)
					Total	Mummified	Stillborn	Live		
G1	JJ16LOM-YJK08	102	65	113	8	6/6	2/2	-	8/8 (100)	19–28.5
		34-5053	66	114	15	0/3	11/11	*** 1/1	12/15 (80)	5–28
G2	JJ16LOM-YYM02	Ll-6061	65	115	14	5/5	6/6	0/3	11/14 (78.5)	6.5–27.5
		62-0093	65	114	18	3/6	9/9	0/3	12/18 (66.6)	17–30
G3	16LOM-KM00	55-4424	65	114	7	7/7	-	-	7/7 (100)	11.5–17.5
		45-0502	64	113	6	5/5	-	0/1	5/6 (83.3)	16.5–28
G4	Control	720	65	114	17	-	0/2	0/15	0/17 (0)	25–30

* Inoculation concentration: 10^3.5^TCID_50_/ml, 2ml/dose. ** Number positive for antigen by qRT-PCR. *** Antigen detection in the blood of a live neonatal suckling piglet.

**Table 3:** Detection of classical swine fever virus (CSFV) RNA in specific pathogen-free (SPF) pigs inoculated with the MLV-LOM or Jeju LOM strains.

Group	Inoculum Strain	No. of SPF Pigs	Sample	No. of CSFV RNA Positive Pigs/No. of Pigs Tested					
				* DPI0	DPI2	DPI4	DPI7	DPI10	DPI14
G1	JJ16LOM-YJK08	10	Blood	0/10	0/10	0/10	** 6/10	8/10	3/10
			Nasal	0/10	0/10	0/10	0/10	0/10	0/10
			Rectal	0/10	0/10	0/10	0/10	0/10	0/10
G2	JJ16LOM-YJK08-F	5	Blood	0/5	0/5	0/5	4/5	5/5	2/5
			Nasal	0/5	0/5	0/5	0/5	0/5	0/5
			Rectal	0/5	0/5	0/5	0/5	0/5	0/5
G3	16LOM-KM00	5	Blood	0/5	0/5	0/5	0/5	2/5	0/5
			Nasal	0/5	0/5	0/5	0/5	0/5	0/5
			Rectal	0/5	0/5	0/5	0/5	0/5	0/5
G4	Control	2	Blood	0/2	0/2	0/2	0/2	0/2	0/2
			Nasal	0/2	0/2	0/2	0/2	0/2	0/2
			Rectal	0/2	0/2	0/2	0/2	0/2	0/2

* DPI: days post-inoculation. ** CSFV RNA positive SPF pigs by the qRT-PCR.

**Table 4:** Organs testing antigen positive after autopsy for SPF pigs inoculated with the MLV-LOM or Jeju LOM strains.

Group	Inoculum Strain	No. of SPF Pigs	Autopsy Day (DPI)	Antigen in Organs * of SPF Pigs (Copy Number (Log _10_) by qRT-PCR/Score by IHC)											
				To	Sp	Lu	Ki	He	Li	Bl	Il	ML	SL	IL	Br
G1	JJ16LOM-YJK08	1	14	2.1/+	1.8/−	-	-	-	-	-	1.9/−	-	2.2/+	-	-
		2	14	2.5/++	2.0/−	-	-	-	-	-	1.4/−	1.6/−	1.8/−	-	-
		3	14	2.0/++	1.8/+	-	-	-	-	-	1.8/+	-	2.4/+	-	-
		4	14	2.3/+	-	-	-	-	-	-	1.7/−	-	1.8/−	-	-
		5	14	2.6/−	-	-	-	-	-	-	-	-	-	-	-
		6	21	-	-	-	-	-	-	-	-	-	-	-	-
		7	21	1.8/+	-	-	-	-	-	-	-	-	-	-	-
		8	21	-	-	-	-	-	-	-	-	-	-	-	-
		9	21	1.6/+	-	-	-	-	-	-	2.2/−	-	-	-	-
		10	21	2.7/+	-	-	-	-	-	2.0/V	-	1.6/−	-	-	-
G2	JJ16LOM-YJK08-F	1	21	-	-	-	-	-	-	-	-	-	-	-	-
		2	21	1.2/+	1.5/−	-	-	-	-	-	-	-	2.0/−	2.6/−	-
		3	21	-	-	-	-	1.8/−	-	-	-	-	-	-	-
		4	21	1.8/+	-	-	-	-	-	-	-	-	2.2/−	-	-
		5	21	2.3/+	-	-	-	-	-	-	-	-	1.9/−	-	-
G3	16LOM-KM00	1	14	1.8/+	1.9/−	-	-	-	-	-	1.7/−	-	1.6/-	-	-
		2	14	2.6/+	1.8/+	-	-	-	-	-	2.1/+	1.8/−	-	-	-
		3	14	2.4/+	1.3/+	-	-	-	-	-	-	-	-	-	-
		4	14	2.1/++	-	-	-	-	-	-	-	1.5/−	-	-	-
		5	14	2.7/+	-	-	-	-	-	-	-	-	-	-	-
G4	Control	1	14	-	-	-	-	-	-	-	-	-	-	-	-
		2	21	-	-	-	-	-	-	-	-	-	-	-	-

* To: tonsil, Sp: spleen, Lu: lung, Ki: kidney, He: heart, Li: liver, Bl: bladder, Il: ileum, ML: mesenteric lymph node, SL: submandibular lymph node, IL: inguinal lymph node, Br: brain.
